# Serum Galectin‐1, Galectin‐9, and YKL‐40 levels in bipolar disorder and their relationship with cognitive functions

**DOI:** 10.1002/brb3.3421

**Published:** 2024-02-12

**Authors:** Elcicek Selahaddin, Demir Bahadir, Sahin Sengul, Taysi Seyithan, Ulusal Hasan, Elboga Gülcin, Altindag Abdurrahman

**Affiliations:** ^1^ Faculty of Medicine Department of Psychiatry Gaziantep University Gaziantep Turkey; ^2^ Faculty of Medicine Department of Biochemistry Gaziantep University Gaziantep Turkey

**Keywords:** bipolar disorder, cognitive functions, Galectin‐1, Galectin‐9, inflammation, neuroinflammation, YKL‐40

## Abstract

**Purpose:**

The number of studies conducted on the role of neuroinflammation in the etiopathogenesis of bipolar disorder has been increasing in recent years. The role of Galectin‐1, Galectin‐9, and YKL‐40, which are considered to play roles in neuroinflammation and the etiopathogenesis of bipolar disorder, and the relationship of these parameters with cognitive functions were investigated in the present study.

**Method:**

Serum Galectin‐1, Galectin‐9, and YKL‐40 levels were measured with the ELISA Method in 64 bipolar euthymic patients and 64 healthy controls. The Stroop and trail‐making tests were administered to assess cognitive functions in all participants.

**Results:**

Serum Galectin‐1, Galectin‐9, and YKL‐40 levels were statistically and significantly lower in the patient group when compared to the healthy control group. The scores of the Stroop test and trail‐making tests were statistically higher in the patient group than in the healthy control group. There was a weak and positive correlation between serum Galectin‐1, Galectin‐9, and YKL‐40 levels and cognitive performance in all participants.

**Discussion and conclusion:**

Statistically significant low levels of serum Galectin‐1, Galectin‐9, and YKL‐40 detected in the patient group suggest that these parameters have important roles in neuroinflammation. The statistically higher Stroop and trail‐making test scores of the patient group compared to the control group indicates that the cognitive performance of the patient group was weaker. Also, the positive correlation between Galectin‐1, Galectin‐9, and YKL‐40 levels and cognitive performance suggests that these molecules may have a neuroprotective role. We think that the present study will contribute to this field where there is very limited data in the literature.

## Abstract 2

There are studies reporting that neuroinflammation plays a role in the etiopathogenesis of bipolar disorder. We examined molecules associated with inflammation that have not been studied before in bipolar disorder and their relationship with cognition.

## Graphical abstract

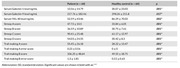

## 1 INTRODUCTION

Bipolar disorder is a recurrent and chronic psychiatric disorder that can significantly impair the functionality of the individual with manic/hypomanic and depressive episodes (Kaplan, 2016). There are studies in the literature reporting that immune system dysfunctions, which cause neuroinflammation are involved in the etiopathogenesis of bipolar disorder (Rosenblat & McIntyre, 2015). The activation of microglia, which play important roles in the Central Nervous System (CNS) immunity, is considered a part of systemic activation of the mononuclear phagocyte system, and systemic inflammation seems to be associated with the inflammation of the CNS (Beumer et al., 2012). It is affected not only by regional inflammation but also by systemic inflammation of the CNS and is associated with the increased incidence of autoimmune/inflammatory diseases in many neuropsychiatric disorders, both in patients and in their first‐degree relatives. The CNS is affected by inflammatory conditions, and inflammation seems to be a common pathway in psychiatric and other medical disease comorbidities (Eaton et al., 2010).

Based on the hypothesis that immunological modulation may be involved in the mood‐stabilizing effect of lithium, the idea that the immune system might have roles in the development of bipolar disorder has been put forward (Horrobin & Lieb, 1981). Previous studies showed that both manic and depressive episodes of bipolar disorder are accompanied by increased acute phase proteins, high levels of proinflammatory cytokines, and activation of neuroinflammatory pathways (Bezchlibnyk et al., 2001; Chen et al., 2019; Naaldijk et al., 2016). In a postmortem study that was conducted to investigate bipolar disorder, the markers showing microglial activity and IL‐1R receptor cascade activity were measured at high levels in the frontal cortex of patients (Rao et al., 2010). Also, cytokines, which are the markers of microglial activity, were found at high levels in the Cerebrospinal Fluid (CSF) samples of these patients even during euthymic periods (Söderlund et al., 2011). In a study that was conducted on a group of bipolar patients who had attempted suicide, increased IL‐6 levels and decreased monoamine activity were shown in CSF samples. It was also observed that this was correlated with the severity of depressive symptoms (Lindqvist et al., 2009).

As members of the lectin family, Galectins play important roles in many immunological processes such as regulation of cell growth, inhibition of apoptosis, induction of apoptosis, regulation of cell adhesion, induction of cell migration, angiogenesis, cancer progression, and inflammation (Rabinovich et al., 2002; van den Brûle et al., 2002). On the other hand, YKL‐40, which is also known as Cutinase 3‐like protein 1 (CHI3L1), or human cartilage glycoprotein 39 (HC‐gp39), was first described in 1989 in a study conducted on new bone proteins as a 40 kDa glycoprotein taking its name from its molecular weight and its N‐terminal last three amino acids, tyrosine, lysine, and leucine (Johansen et al., 1992). It was reported that YKL‐40, which is expressed by many cells, including macrophages, chondrocytes, neutrophils, and synovial fibroblasts, plays active roles in acute and chronic inflammation, especially in the remodeling of the extracellular matrix (Johansen, 2006; Rehli et al., 2003).

In the literature, there is plenty of evidence that patients have permanent cognitive deficits in areas related to attention, executive functions, and verbal memory, even in the euthymic phase of bipolar disorder (Martínez‐Arán et al., 2004; Thompson et al., 2005; van Gorp et al., 1998). Studies show that cognitive deficits are mostly in areas such as verbal memory and executive functions in bipolar patients (Arts et al., 2008; Lloyd et al., 2009).

The present study aimed to show the roles of Galectin‐1, Galectin‐9, and YKL‐40, which are considered to have roles in neuroinflammation, the etiopathogenesis of bipolar disorder, and the relationship of these parameters with cognitive functions.

## 2 METHOD

For the present study, which was conducted in the prospective paired case‐control design, approval was obtained from Gaziantep University Ethics Committee with the decision number 2021/212 on 27.08.2021. A total of 64 euthymic patients, who were diagnosed with bipolar disorder according to DSM‐5 diagnostic criteria or followed up with this diagnosis, without any additional diagnosis, who applied to Gaziantep University Mental Health and Diseases Clinic between September 2021 and February 2022, were included in the study. The patient group consists of bipolar disorder‐1 (*n*:45) and bipolar disorder‐2 (*n*:19) patients. Also, 64 healthy controls without any disease, matched according to gender, age, body mass index (BMI), smoking, and educational status, were recruited for the study. The informed consent form was read and signed by all the participants.

Young Mania Rating Scale (YMRS), which was developed by Young to measure the severity of mania, was adapted into Turkish by Karadağ et al. (2001) by conducting a validity and reliability study. The Hamilton Depression Rating Scale (HDRS), which is used in the evaluation of depression, whose validity and reliability study was conducted by Akdemir et al. (1996) by adapting it into Turkish, was also used in the study.

The Sociodemographic Data Form was filled out for all participants. The YMRS and HDRS were applied to the participants to identify manic and depressive episodes.

The patient participation was determined for at least 8 weeks with YMRS < 7 and HDRS < 6 for the euthymic period. Stroop and Trail‐Making Tests were administered to all participants to evaluate their cognitive performances. Blood samples were taken from all participants in the morning after fasting for at least 8 hours and sent to the biochemistry laboratory and stored at −80°C until the study day in centrifuged form. The measurement of Galectin‐1 (BT LAB, Cat. No: E2989Hu), Galectin‐9 (BT LAB, Cat. No: E2998Hu), and YKL‐40 (LAB, Cat. No: E2063Hu) levels was made according to the ELISA Method. The results were measured in nanogram/milliliter (ng/mL).

The Stroop test, which is a neuropsychological test that reflects the activity of the frontal region, was developed by Stroop in 1935 to evaluate the ease of changing the perceptual setup in line with changing demands and under the influence of a disturbance, the ability to suppress a habitual behavior pattern and perform an unusual behavior (Spreen & Strauss, 1998). The completion times of sections A, B, and C of the Stroop test, whose Turkish validity and reliability study was conducted by Karakaş et al. (1999), were recorded as the test scores. If the participant made errors or corrections, their number was also added to these scores. The Stroop D Score, which measures the ability to ignore disruptive stimuli (interference), was obtained by subtracting the Stroop B Score from the Stroop C Score. The Trail‐Making Test, whose Turkish validity and reliability study was conducted by Türkeş et al. (2015), was used to evaluate the executive functions such as processing speed based on visual scanning ability, changing setup between sets of stimuli, and following sequentiality. The duration of the participant to complete the test was recorded as the test score. If the participant made an error or correction, their number was recorded as an error score.

The conformity of the numerical variables to the normal distribution was tested with the Shapiro–Wilk Test. The Mann–Whitney *U* test was used to compare the nonnormally distributed variables in two groups. Logistic regression analysis with adjustments for age, gender, and education was performed to examine the effects on cognitive functions and blood parameters between bipolar disorder patients and controls. The relationships between nonnormally distributed numerical variables were tested with the Spearman's rank correlation coefficient. The relationships between categorical variables were tested with the chi‐square test. The SPSS 22.0 Windows version package program was used in the analysis and *p* < .05 was considered significant.

## 3 RESULTS

The sociodemographic characteristics of the study group, which consisted of 64 euthymic bipolar patients and 64 healthy controls, are given in **Table** **1**. No statistically significant differences were detected between the patient and control group in terms of mean age, gender distribution, marital status, education level, smoking status and BMI (*p* = .211, *p* = .216, *p* = .172, *p* = .534, *p* = .717, *p* = .176, respectively).

**TABLE 1:** The comparison of the sociodemographic data of the groups.

		Group		
		Patient	Healthy control	
		Number, *n* (%)	Number, *n* (%)	*p* Value
Age, years (mean ± SD)		33.2 ± 8.78	31.41 ± 6.93	.211
Gender	**Male**	29 (45.3)	36 (56.3)	.216
	**Female**	35 (54.7)	28 (43.8)	
Marital status	**Single**	28 (43.8)	26 (40.6)	.172
	**Married**	31 (48.4)	37 (57.8)	
	**Divorced‐widow(er)**	5 (7.8)	1 (1.6)	
Educational status	**Primary school**	6 (9.4)	3 (4.7)	.534
	**High school**	22 (34.4)	21 (32.8)	
	**University**	36 (56.3)	40 (62.5)	
Smoking status	**Nonsmoking**	40 (62.5)	38 (59.4)	.717
	**Smoking**	24 (37.5)	26 (40.6)	
BMI (mean ± SD)		24.86 ± 2.75	24.23 ± 2.37	.176

Abbreviation: SD, standard deviation.

Serum Galectin‐1 and Galectin‐9 levels were statistically and significantly lower in the patients than in the healthy control group (*p* = .001 and *p* = .017, respectively). Similarly, the mean serum YKL‐40 level was significantly lower in the patients compared to the participants in the healthy control group (*p* = .002). We performed regression analysis to investigate the potential influence of confounding factors (age, sex, and BMI) on the observed significant differences in serum Galectin‐1, Galectin‐9, and YKL‐40 levels between patient and control groups (*p* < .001 β = .046, *p* = .028 β = .003, and *p* = .04 β = .008 respectively).

The scores of the bipolar patients in all Stroop A, B, C, and D tests were statistically and significantly higher than the healthy control group (*p* = .001 for all tests). We conducted regression analysis with adjustments for age, sex, and education to examine their impact on cognitive function between bipolar disorder patients and controls. Stroop A Score, Trail‐making A Score, and Trail‐making B Score remained significantly different between the groups (*p* = .002 β = −.306, *p* = .009 β = −.079, and *p* = .016 β = −.064, respectively), while other cognitive function test outcomes lost significance. In summary, Stroop A, Trail‐making A Score, and Trail‐making B Score were identified as meaningful predictors of cognitive function in bipolar patients following the specified adjustments.

When the trail‐making test results were evaluated, the A and B scores of the bipolar patients were similarly higher than the healthy controls at significant levels (*p* = .001 for both tests). The error scores of the trail‐making A and B tests were also higher in the patient group at significant levels (*p* = .001 for both tests). The comparison results of the clinical characteristics of the groups are given in **Table** **2**.

**TABLE 2:** The comparison of the biochemical and cognitive test results of the groups.

	Patient (*n* = 64)	Healthy control (*n* = 64)	*p*
Serum Galectin‐1 level ng/mL	13.56 ± 14.79	30.07 ± 23.03	.001*
Serum Galectin‐9 level ng/mL	217.76 ± 182.46	298.26 ± 211.8	.017*
Serum YKL‐40 level ng/mL	52.59 ± 43.46	86.29 ± 70.03	.002*
Stroop A score	47.72 ± 10.5	33.84 ± 6.05	.001*
Stroop B score	36.59 ± 10.89	30.75 ± 7.61	.001*
Stroop C score	90.41 ± 25.48	61.17 ± 12.97	.001*
Stroop D score	54.03 ± 24.55	30.42 ± 8.3	.001*
Trail‐making A score	55.45 ± 26.18	28.22 ± 13.47	.001*
Trail‐making A error score	0.23 ± 0.56	0 ± 0	.001*
Trail‐making B score	106.25 ± 48.64	49.55 ± 18.75	.001*
Trail‐making B error score	1.3 ± 1.81	0.22 ± 0.63	.001*

Abbreviation: SD, standard deviation. Significant values are shown in bold with an “*”

In the correlation analysis, a strong negative relationship was found between age and education level (*r* = −.437, *p* < .001). There was a positive correlation between age and Stroop A, B, C, trail‐making A, B scores (*r* = .224, *p* = .001, *r* = .296, *p* = .001, *r* = .206, *p* = .001, *r* = .190, *p* = .003, *r* = .202, *p* = .002, respectively). There was a strong positive correlation between age and BMI (*r* = .863, *p* < .001). There was a strong negative correlation between educational status and Stroop A, B, C, trail‐making A, B scores (*r* = −.263, *p* = .003, *r* = −.424, *p* < .001, *r* = −.274, *p* = .002, *r* = −.393, *p* < .001, *r* = −.264, *p* = .003, respectively). There was a positive correlation between age of disease onset and smoking and status and BMI (*r* = .313, *p* = .001, *r* = .496, *p* < .001, respectively). There was a positive correlation between duration of disease and number of manic episodes, depressive episodes and BMI (*r* = .686, *p* < .001, *r* = .710, *p* < .001, *r* = 652, *p* < .001, respectively).

A correlation analysis was made to evaluate the relationship between serum parameters (Galectin‐1, Galectin‐9, and YKL‐40) in patient and control groups, cognitive tests (Stroop A, B, C, D scores and trail‐making A, B tests), and other clinical characteristics analysis. According to this correlation analysis, it was found that serum Galectin‐1 level and the age of onset of the disease showed a weak and negative correlation (*r* = −.248, *p* = .048). There was also a weak and negative correlation between serum Galectin‐1 level and Stroop A, B, C, and D levels ((*r* = −.366, *p* < .001, *r* = −.310, *p* < .001, *r* = −.383, *p* < .001, *r* = −.251, *p* = .004, respectively). Similarly, a weak and negative correlation was detected between serum Galectin‐1 level, and trail‐making A score, trail‐making A error score, and trail‐making B score (*r* = −.391, *p* < .001, *r* = −.241, *p* = .006, *r* = −.367, *p* < .001, respectively).

A strong and positive correlation was detected between serum Galectin‐9 level and serum YKL‐40 level (*r* = .531, *p* < .001). On the other hand, there was a weak and negative correlation between serum Galectin‐9 level and cognitive tests Stroop A, Stroop C, and Stroop D scores and trail‐making A, trail‐making A error score, trail‐making B and trail‐making B error scores (*r* = −.250, *p* = .004, *r* = −.220, *p* = .012, *r* = −.233, *p* = .008, *r* = −.194, *p* = .028, *r* = −.261, *p* = .003, *r* = −.234, *p* = .008, *r* = −.176, *p* = .047, respectively).

The serum YKL‐40 levels also had a weak and negative correlation with Stroop A, B, and C scores (*r* = −.208, *p* = .019, *r* = −.249, *p* = .005, *r* = −.300, *p* = .001, respectively). Similarly, a negative correlation was detected between YKL‐40 levels and trail‐making A score, trail‐making A error score, and trail‐making B score (*r* = −.274, *p* = .002, *r* = −.217, *p* = .014, respectively, *r* = −.221, *p* = .012) (the data are shown in Table 3).

**TABLE 3:** The correlation analysis of the relationship between serum Galectin‐1, Galectin‐9, and YKL‐40 levels and cognitive tests in all participants.

		Stroop A Score	Stroop B Score	Stroop C Score	Stroop D Score	Trail‐making A Score	Trail‐making A error Score	Trail‐making B Score	Trail‐making B error Score
Serum Galectin‐1 level	***r***	–.366^**^	–.310^**^	–.383^**^	–.251^**^	–.391^**^	–.241^**^	–.367^**^	–.082
	***p***	**.000**	**.000**	**.000**	**.004**	**.000**	**.006**	**.000**	.357
	***n***	128	128	128	128	128	128	128	128
Serum Galectin‐9 level	***r***	–.250^**^	**–.075**	–.220^**^	–.233^**^	–.194^**^	–.261^**^	–.234^**^	–.176^*^
	***p***	**.004**	**.399**	**.012**	**.008**	**.028**	**.003**	**.008**	**.047**
	***n***	128	128	128	128	128	128	128	128
Serum YKL‐40 level	***r***	–.208^**^	**–.029**	–.249^**^	–.300^**^	–.274^**^	–.217^**^	–.221^**^	–.052
	***p***	**.019**	**.742**	**.005**	**.001**	**.002**	**.014**	**.012**	.560
	***n***	128	128	128	128	128	128	128	128

*Note*: The Spearman's test was used in the correlation analysis. *r*: correlation coefficient, *p*: significance value, *n*: number of participants. Significant values are shown in bold with an “**.” This article is the article of a psychiatry specialist thesis.

## 4 DISCUSSION

In the present study that was conducted to evaluate serum Galectin‐1, Galectin‐9, and YKL‐40 levels in patients with bipolar disorder, compare them with healthy controls, and test the relationship with cognitive functions, serum Galectin‐1 levels were found to be significantly lower in patients with bipolar disorder. It was also found that cognitive performance increased as serum Galectin‐1 level increased. It was determined in the correlation analysis that the patients diagnosed at an earlier age had higher serum Galectin‐1 levels.

Studies show that immune system dysfunctions that cause neuroinflammation play a role in the etiopathogenesis of bipolar disorder, as in many chronic psychiatric disorders (Rosenblat & McIntyre, 2015). To the best of our knowledge, there are studies in the literature investigating the relationship of Galectin‐1 with neurological diseases and with schizophrenia, whose relationship with bipolar disorder has not been investigated before, to a limited extent. It was reported that the expression of Galectin‐1, which was reported to have a neuroprotective effect by playing roles in axonal regeneration (Egnaczyk et al., 2003), changes in many neurological diseases and is associated with neuroregeneration (McGraw et al., 2005). It was reported in a previous study that investigated the roles of Galectin‐1 in HIV neuropathogenesis that Galectin‐1 suppresses neuroinflammation playing neuroprotective roles (Reynolds et al., 2012). Similarly, it was reported in the study of Aalinkeel et al. that Galectin‐1 regulates microglial functions in vitro by decreasing the release of proinflammatory cytokines and increasing the release of anti‐inflammatory cytokines also suppressing the proliferation of astrocytes, reducing astrogliosis and playing neuroprotective roles (Aalinkeel et al., 2017). In another study conducted with Parkinson's patients, the CSF Galectin‐1 levels of the patients were found to be quite low when compared to the control group, and as a result, it was considered that Galectin‐1 could be a potential biomarker for Parkinson's patients (Marques et al., 2019). In a study conducted by Wada et al. (2003) with ALS patients with Amyotrophic Lateral Sclerosis (ALS), it was concluded that Galectin‐1 immunoreactivity was decreased in the skin of patients, which suggested that cutaneous Galectin‐1 is involved in the pathogenesis of ALS. In a previous study investigating the role of neuroinflammation in schizophrenia, no statistically significant differences were detected regarding the Galectin‐1 levels in patients with schizophrenia when compared to healthy controls. As a result of the study, it was reported that Galectin‐1 levels were induced by activated astrocytes and such a result was found in schizophrenia because there were no obvious pathological changes such as astrocytic gliosis (Kajitani et al., 2017). In the study of Yüksel et al. (2020), who examined the roles of inflammation in schizophrenia, Galectin‐1 levels were found to be higher in unaffected siblings of schizophrenic patients when compared to patients themselves and healthy controls. According to the authors, elevated Galectin‐1 levels in unaffected siblings led to the conclusion that Galectin‐1 had protective roles against inflammation (Yüksel et al., 2020). In light of all these data, it can be argued that Galectin‐1 has neuroprotective roles against inflammation. In the present study, it was found that the Galectin‐1 levels of the patients were lower than the healthy controls, which was consistent with the literature data. The weak and positive correlation between Galectin‐1 level and cognitive performance in the present study is also a finding supporting the neuroprotective roles of Galectin‐1. The negative correlation of serum Galectin‐1 level with the age of onset of the disease in patients also suggests that Galectin‐1 has neuroprotective roles. The decreased serum Galectin‐1 levels in the preclinical period might have caused the neuroprotective defense to be insufficient and caused the emergence of the disease; however, longitudinal follow‐up studies are needed to support this view more strongly.

In the present study, it was also found that serum Galectin‐9 levels were statistically and significantly lower in the bipolar patient group than in the healthy control group. A weak and positive correlation was found between serum Galectin‐9 levels and cognitive performance in all participants. To the best of our knowledge, there is no study in the literature investigating the relationship between Galectin‐9 and psychiatric diseases. There are a limited number of studies investigating the relationship between Galectin‐9 and neurological diseases. In a previous study conducted with HIV‐infected patients, increased CSF Galectin‐9 levels were found to be associated with CNS immune activation and impaired cognitive performance in elderly patients. CSF Galectin‐9 levels of patients with HIV‐related dementia or mild cognitive impairment in elderly patients were found to be higher than those with normal cognitive performance in this study, which did not have a statistically significant result when young patients were compared (Premeaux et al., 2019). These results, which are inconsistent with the results found in the present study, might have occurred because the study was conducted in CSF or the type of disease studied or by the laboratory methods used. In a study conducted by Burman and Svenningsson (2016) with patients who had Multiple Sclerosis (MS), the CSF Galectin‐9 concentration of Secondary Progressive Multiple Sclerosis (SPMS) patients was found to be higher than that of Relapsing‐Remitting Multiple Sclerosis (RRMS) patients and healthy controls. Although no statistical differences were detected between RRMS patients and the control group in a cohort of the study, CSF Galectin‐9 levels of RRMS patients in the other cohort were found to be higher in the control group. Also, in this study, CSF Galectin‐9 levels were not found to be associated with inflammation parameters (Burman & Svenningsson, 2016). As a result of a cohort, no significant differences were detected between RRMS patients and healthy control groups in terms of CSF Galectin‐9 levels, and in addition, the lack of a correlation between CSF Galectin‐9 levels and inflammation parameters in this study might explain the conflict with the results found in the present study. In light of these data, it is suggested that Galectin‐9 plays a neuroprotective role in the etiopathogenesis of bipolar disorder, it must be noted that this hypothesis needs support with further studies. To the best of our knowledge, the present study is important because it is the first study conducted on this subject.

It was found in the present study that the serum YKL‐40 levels were statistically and significantly lower in the patient group when compared to the healthy control group. It was also found that there was a weak and positive correlation between serum YKL‐40 level and cognitive performance in all participants. A moderately strong and positive correlation was detected between serum YKL‐40 level and serum Galectin‐9 level in all participants. There are studies in the literature investigating the relationship between neuropsychiatric diseases and YKL‐40. In a study conducted with Alzheimer's patients, it was stated that CSF YKL‐40 levels were higher than cognitively normal individuals (Groblewska & Mroczko, 2017). In the literature, there is a limited number of studies investigating the relationship between YKL‐40 and bipolar disorder. In a study conducted by Jakobsson et al. (2015) in a bipolar euthymic patient group, it was found that CSF and serum YKL‐40 levels were higher in the patient group than in the healthy control group. In another study investigating the relationship between peripheral inflammatory markers and decreased brain volume in bipolar‐1 patients, high YKL‐40 levels were associated with decreased left anterior cingulum, left frontal lobe, right superior temporal gyrus, and supramarginal gyrus volumes (Tsai et al., 2021). In a study that was conducted by Isgren et al. (2017) on CSF YKL‐40 levels, it was reported that a negative correlation was detected between the occurrence of manic/hypomanic episodes and psychotic symptoms. CSF YKL‐40 levels were found to be associated with impaired executive functions in the euthymic period of bipolar disorder in a previous study that investigated the relationship between neuroinflammation and permanent cognitive impairment in the euthymic period of bipolar disorder (Rolstad et al., 2015). Isgren et al. (2017) found a negative correlation between CSF YKL‐40 levels and the onset of manic/hypomanic episodes and psychotic symptoms, indicating that YKL‐40 has neuroprotective roles and is consistent with our results. However, it is seen that the results of the other studies mentioned above are inconsistent with the results found in the present study. This may have been because the present study was conducted in sera rather than CSF, the clinical characteristics of the groups included in other studies were different, ethnicity or the laboratory methods used. In this sense, it seems that more studies are needed to elucidate the relationship between bipolar disorder and YKL‐40.

The cognitive performance of the patient group was found to be statistically and significantly lower than the control group in the study. We also found a weak positive correlation between galectin‐1, galectin‐9 and YKL‐40 serum levels, which we think may be neuroprotective, and cognitive performance. This is also another result supporting the view that these molecules have neuroprotective roles. There are many studies in the literature reporting that the cognitive performance of euthymic bipolar patients is worse than that of healthy controls. Thompson et al. (2005) reported that bipolar patients in remission have permanent cognitive deficits. Similarly, it was reported in another study that cognitive dysfunction in bipolar disorder persists in the euthymic period of the disease, and there are deficits in verbal memory and executive functions in healthy first‐degree relatives of bipolar patients (Robinson et al., 2006). In a study conducted by Martinez‐Aran et al. (2007), it was reported that the Stroop and trail‐making tests performances of bipolar patients were worse than those of healthy controls, and the cognitive deficit in bipolar disorder was especially in verbal memory and executive functions. In light of these data, it is possible to argue that our finding that bipolar euthymic patients have worse cognitive performance when compared to healthy controls is consistent with the literature data.

The study had some limitations. First, its cross‐sectional design was the main limitation. In a longitudinal study, examining the course of these molecules with the episodes of the disease will make significant contributions to the literature in this respect. Also, the fact that the study was conducted in serum rather than CSF can be considered another limitation. On the other hand, it can be thought that the treatments received by the patients may also affect the results. Investigating the CSF levels of these immune system elements, which we think are associated with neuroinflammation, may provide more information on this subject.

## 5 CONCLUSION

As a result of the present study, it is possible to argue that Galectin‐1, Galectin‐9, and YKL‐40, which we think have neuroprotective roles, are also critical in neuroinflammatory processes that play a role in the etiopathogenesis of bipolar disorder. If supported with future studies, we think that Galectin‐1, Galectin‐9, and YKL‐40 can be used as a marker in the diagnosis and follow‐up of patients who do not meet the diagnostic criteria for bipolar disorder.

## AUTHOR CONTRIBUTIONS

DB and ES contributed to the searched the literature and designed the study; DB, ES, SS, TS, and UH contributed to the acquisition of data; TS and UH performed the biochemical examinations; DB, ES, SS, EG and AA assisted in the data processing and performed the functional analysis; DB and ES reviewed the data and drafted the manuscript. All authors have read and approved the final manuscript.

## CONFLICT OF INTEREST STATEMENT

All of the authors declare that there are no conflicts of interest in connection with this paper.

### FUNDING

There is no funding source for this research.

### PEER REVIEW

The peer review history for this article is available at https://publons.com/publon/10.1002/brb3.3421.

## Data Availability

The datasets generated and/or analyzed during the current study are available from the corresponding author upon reasonable request.
